# Factors that influence treatment delay in patients with colorectal cancer

**DOI:** 10.18632/oncotarget.13574

**Published:** 2016-11-24

**Authors:** Irene Zarcos-Pedrinaci, Alberto Fernández-López, Teresa Téllez, Francisco Rivas-Ruiz, Antonio Rueda A, María Manuela Morales Suarez-Varela, Eduardo Briones, Marisa Baré, Antonio Escobar, Cristina Sarasqueta, Nerea Fernández de Larrea, Urko Aguirre, José María Quintana, Maximino Redondo

**Affiliations:** ^1^ Research Unit, Agencia Sanitaria Costa del Sol, Marbella, Spain; ^2^ Servicio de Cirugía, Agencia Sanitaria Costa del Sol, Marbella, Spain; ^3^ Servicio de Oncología Médica, Agencia Sanitaria Costa del Sol, Marbella, Spain; ^4^ Unit of Public Health, Hygiene and Environmental Health, Department of Preventive Medicine and Public Health, Food Science, Toxicology and Legal Medicine, University of Valencia, CIBER-Epidemiology and Public Health (CIBERESP), Valencia, Spain; ^5^ Public Health Unit, Distrito Sanitario Sevilla, Consorcio de Investigación Biomédica de Epidemiología y Salud Pública, Madrid, Spain; ^6^ Clinical Epidemiology and Cancer Screening, Corporació Sanitària Parc Taulí, Sabadell, Spain; ^7^ Research Unit, Hospital Universitario Basurto, Bilbao, Spain; ^8^ Research Unit, Donostia University Hospital, San Sebastián, Spain; ^9^ Area of Environmental Epidemiology and Cancer, National Epidemiology Centre, Instituto de Salud Carlos III, Consortium for Biomedical Research in Epidemiology and Public Health (CIBER Epidemiología y Salud Pública, CIBERESP), Madrid, Spain; ^10^ Research Unit, Hospital Galdakao-Usansolo, Galdakao, Spain; ^11^ Red de Investigación en Servicios de Salud en Enfermedades Crónicas – REDISSEC, Spain

**Keywords:** colorectal, cancer, delay, treatment, education

## Abstract

A prospective study was performed of patients diagnosed with colorectal cancer (CRC), distinguishing between colonic and rectal location, to determine the factors that may provoke a delay in the first treatment (DFT) provided.

2749 patients diagnosed with CRC were studied. The study population was recruited between June 2010 and December 2012. DFT is defined as time elapsed between diagnosis and first treatment exceeding 30 days.

Excessive treatment delay was recorded in 65.5% of the cases, and was more prevalent among rectal cancer patients. Independent predictor variables of DFT in colon cancer patients were a low level of education, small tumour, ex-smoker, asymptomatic at diagnosis and following the application of screening. Among rectal cancer patients, the corresponding factors were primary school education and being asymptomatic.

We conclude that treatment delay in CRC patients is affected not only by clinicopathological factors, but also by sociocultural ones. Greater attention should be paid by the healthcare provider to social groups with less formal education, in order to optimise treatment attention.

## INTRODUCTION

Colorectal cancer (CRC) is a major public health problem, with major impact on morbidity and mortality. It is the second most prevalent malignancy worldwide, and is also second in incidence and mortality in most developed countries. In Europe, five-year survival rates are 44-64%, and in Spain the EUROCARE-4 project calculated a survival rate of 61.5% [1]. As a result of population aging, together with diagnostic and therapeutic advances, the number of cancer patients has increased significantly, and this situation is placing great pressure on the cancer care system, reflecting the growing importance of this group of diseases as a public health problem.

Early diagnosis of cancer and hence early treatment is a fundamental objective in cancer care procedures. Although delays attributable to the health system constitute a small proportion of the biological life of a tumour, noticeable hospital delay (from first hospital visit to diagnosis or from diagnosis to treatment) may provoke stress and decrease the patient's quality of life. In fact, delays in initiating treatment are the leading cause of malpractice complaints [2].

While some studies indicate that treatment delay negatively affects the prognosis of patients with cancer, particularly CRC, others have found no such association [3, 4]. Moreover, it has been reported that delay is often attributable to tumour factors such as clinical stage and location, and not only to the health system, such as hospital admission procedures. The impact of treatment delay on survival, and the significance of the diverse factors involved, have yet to be determined [5]. Waiting time is a complex variable, which can reflect the patient's own behaviour, the clinical course, the functioning of the health system and tumour biology [6].

Taking into account the dearth of prospective studies designed to analyse treatment delay, with large cohorts of patients and distinguishing between colonic and rectal tumours, in this study we evaluate the degree to which treatment delay is influenced by the sociodemographic conditions of patients and by the clinical and pathological characteristics of the tumour.

## RESULTS

### Descriptive analysis

During the recruitment period, the 22 participating centres recruited 2,749 patients who met the criteria for inclusion. Of these, 330 (12%) were later excluded from the study because it was not possible to determine the treatment delay. Thus, the final patient sample was composed of 2,419 records. The sociodemographic and clinicopathological characteristics of the study population are shown in Table 1.

**Table 1:** Sociodemographic and clinical characteristics for all cases and segmented by type of tumour

	Total		Colon		Rectal		*p*
	n	%	n	%	n	%	
**Sex**							
Male	1539	63.6	1092	62.2	447	67.3	***0.023***
Female	880	36.4	663	37.8	217	32.7	
**Age**							
*Mean - SD*	*68.3*	±*10.9*	*68.8*	±*10.8*	*66.9*	±*11.0*	***<0.001***
**Marital status^1^**							
Single	150	7.6	100	6.9	50	9.1	***0.048***
Married-Cohabiting	1434	72.2	1028	71.4	406	74.2	
Separated-Divorced	100	5.0	76	5.3	24	4.4	
Widowed	302	15.2	235	16.3	67	12.2	
**Education profile^2^**							
No education-Primary education	1531	77.2	1114	77.1	417	77.2	*1.000*
Secondary-University	453	22.8	330	22.9	123	22.8	
**Currently in work^3^**							
No	1493	76.3	1072	75.3	421	78.7	*0.135*
Yes	465	23.7	351	24.7	114	21.3	
**BMI^4^**							
*Mean - SD*	*27.7*	±*4.8*	*28.0*	±*4.9*	*27.1*	±*4.5*	***<0.001***
**Smoking habit^5^**							
Never	1109	47.8	831	49.6	278	43.3	***0.008***
Current smoker	302	13.0	201	12.0	101	15.7	
Ex-smoker	908	39.2	645	38.5	263	41.0	
**Family history of neoplasias^6^**							
No	1339	61.3	990	63.1	349	56.7	***0.007***
Yes	846	38.7	580	36.9	266	43.3	
**Family history of CRC^7^**							
No	1295	86.2	934	86.3	361	85.7	*0.837*
Yes	208	13.8	148	13.7	60	14.3	
**Specific signs and symptoms^8^**							
Asymptomatic	204	8.8	160	9.6	44	6.9	***<0.001***
Moderate signs and symptoms	381	16.5	300	18.0	81	12.6	
Severe signs and symptoms	1724	74.7	1207	72.4	517	80.5	
**Type of tumour**							
Colon	1755	72.6					
Recto	664	27.4					
**Size of tumour^9^**							
Locally small (T0-T1-T2)	681	28.8	376	21.9	305	47.0	***<0.001***
Locally large (T3-T4)	1686	71.2	1342	78.1	344	53.0	
**Lymph nodes^10^**							
Absent	1464	62.7	1028	60.0	436	70.1	***<0.001***
Present	871	37.3	685	40.0	186	29.9	
**Histological diagnosis^11^**							
Adenocarcinoma	2152	89.6	1555	89.0	597	91.3	*0.121*
Mucinous carcinoma or other types	249	10.4	192	11.0	57	8.7	
**Metastasis^12^**							
Absent	2057	91.9	1483	91.2	574	93.8	*0.056*
Present	181	8.1	143	8.8	38	6.2	
**Differentiation^13^**							
Low grade	1790	86.9	1333	86.4	457	88.2	*0.336*
High grade	270	13.1	209	13.6	61	11.8	
**Vascular invasion^14^**							
Absent	1764	86.4	1259	84.4	505	92.0	***<0.001***
Present	277	13.6	233	15.6	44	8.0	
**Perineural invasion^15^**							
Absent	1627	81.4	1165	80.1	462	84.8	**0.019**
Present	373	18.7	290	19.9	83	15.2	
**Carcinoembryonic antigen (CEA)^16^**							
Normal (0-5)	1328	68.7	917	67.5	411	71.6	*0.083*
Abnormal (>5)	605	31.3	442	32.5	163	28.4	
**Cancer antigen 19-9^17^**							
Normal (1-37)	944	85.4	620	84.1	324	88.0	*0.099*
Abnormal (>37)	161	14.6	117	15.9	44	12.0	
**Prior screening^18^**							
No	1868	80.8	1330	79.1	538	85.4	***0.001***
Yes	443	19.2	351	20.9	92	14.6	

Losses: 1=433; 2=435; 3=461; 4=552; 5=100; 6=234; 7=916; 8=110.

Losses: 9=52; 10=84; 11=18; 12=181; 13=359; 14=378; 15=419; 16=486; 17=1314; 18=108

### Treatment delays and types of treatment

For all tumours, the most common initial treatment was surgery (81.4%), followed by chemotherapy (13%) (p<0.001). For rectal tumours alone, surgery and chemotherapy were also the most common treatment options (40.5% and 39.5%, respectively).

**Figure 1:**
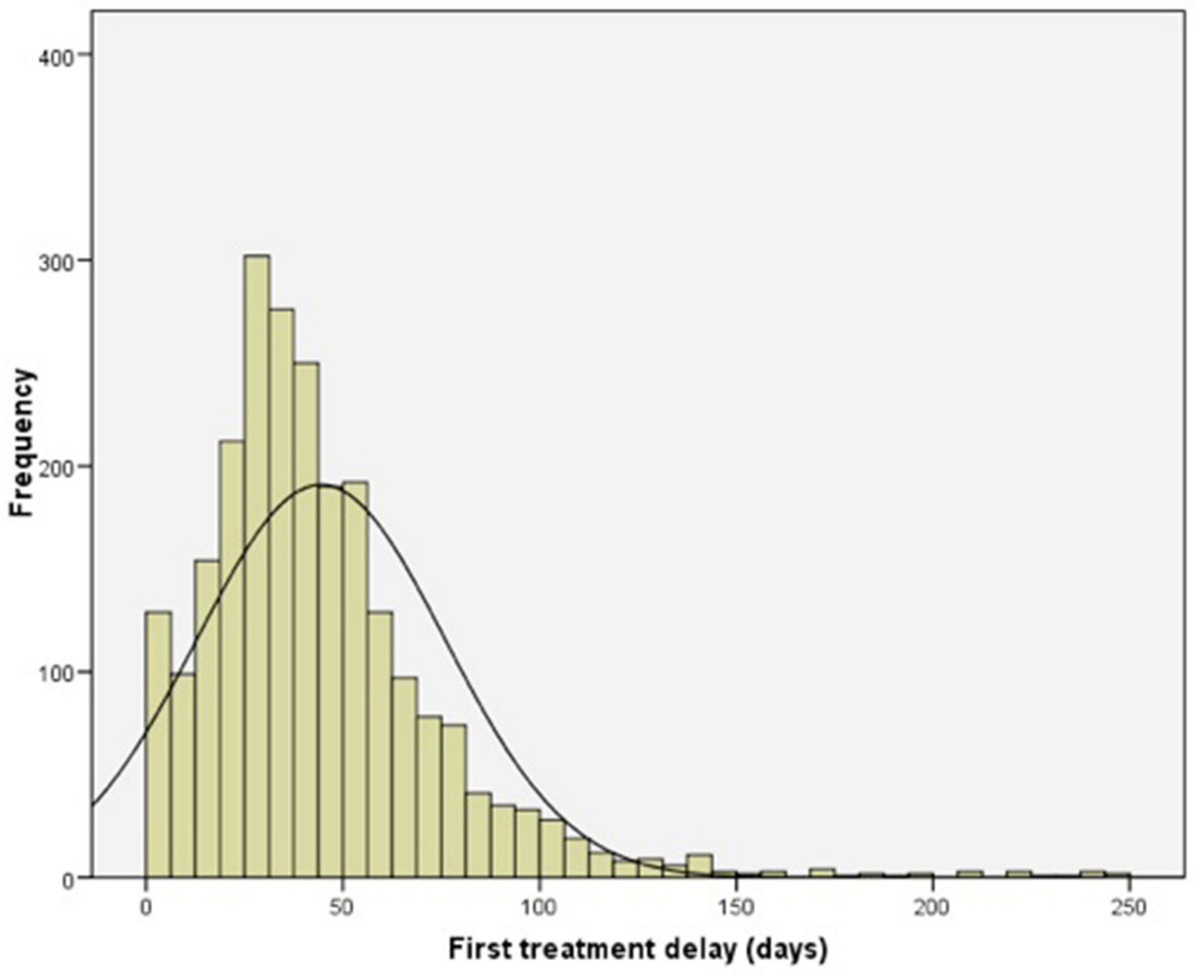
Frequency histogram of delay (in days) to first treatment for patients with CRC

A histogram showing the distribution of treatment delay is shown in Figure 1. A delay to first treatment exceeding 30 days was recorded in 65.5% of cases [95% CI: 63.6-67.4], and this value was higher (p<0.001) for rectal tumours (74.4%) than for colon tumours (62.2%) (Table 2). Stratifying according to the first mode of treatment administered and by tumour location, there was a higher frequency of delay for surgical treatment for rectal tumours than for colon tumours (79.2% vs. 62.2%) (p<0.001). No significant differences were observed for the other treatment strategies.

**Table 2:** Type of first treatment and delays

	Total		Colon		Rectal		*p*
	n	%	n	%	n	%	
**First line of treatment**							
Surgery	1968	81.4	1699	96.8	269	40.5	***<0.001***
Chemotherapy^1^	314	13.0	52	3.0	262	39.5	
Radiotherapy	137	5.7	4	0.2	133	20.0	
**Delay in first treatment**							
≤30 days	834	34.5	664	37.8	170	25.6	***<0.001***
>30 days	1585	65.5	1091	62.2	494	74.4	
**Delay before surgery**							
≤30 days	699	35.5	643	37.8	56	20.8	***<0.001***
>30 days	1269	64.5	1056	62.2	213	79.2	
**Delay before chemotherapy**							
≤30 days	96	30.6	20	38.5	76	29.0	*0.235*
>30 days	218	69.4	32	61.5	186	71.0	
**Delay before radiotherapy**							
≤30 days	39	28.5	1	25.0	38	28.6	*1.000*
>30 days	98	71.5	3	75.0	95	71.4	

^1^ With or without radiotherapy

### Relation between treatment delay and the patients’ sociodemographic and clinicopathological characteristics

In our analysis of the relation between the presence of DFT and each of the sociodemographic variables, those that were significantly associated with greater DFT in patients with cancer of the colon were male sex, low level of education or no formal education, BMI (28±5.1), ex-smoker and asymptomatic at diagnosis. The most relevant tumour characteristics were small local extension and the absence of nodes, of metastasis and of perineural invasion. Treatment delays in patients with tumours presenting normal values for carcinoembryonic antigen and for cancer antigen 19-9 were greater than among patients presenting abnormal values for these parameters. Finally, the treatment delay in patients who had received prior screening was greater than among those who had not had this test (Table 3). For rectal tumours, the variables that were significantly related to a higher level of DFT were primary studies or no formal education, being asymptomatic and having had prior screening (Table 4).

**Table 3:** Bivariate and multivariate analysis with DFT in patients with colon cancer

	≤30 days		>30 days		*Crude analysis*		Adjusted analysis*	
	n	%	n	%	p	OR 95% CI	p	OR 95% CI
**Sex**								
Male	393	36.0	699	64.0	***0.041***	***1.00***		
Female	271	40.9	392	59.1		***0.81 [0.67-0.99]***		
**Age**								
*Mean - SD*	68.8	±11.3	68.8	±10.4	*0.915*	*1.00 [0.99-1.01]*		
**Marital status**								
Single	38	38.0	62	62.0	*0.182*	*1.00*		
Married-Cohabiting	374	36.4	654	63.6		*1.07 [0.70-1.64]*		
Separated-Divorced	21	27.6	55	72.4		*1.60 [0.84-3.06]*		
Widowed	97	41.3	138	58.7		*0.87 [0.54-1.41]*		
**Education profile**								
No education-Primary education	388	34.8	726	65.2	***0.001***	*1.00*	***0.008***	*1.00*
Secondary-University	148	44.8	182	55.2		***0.66 [0.51-0.84]***		***0.69[0.52-0.91]***
**Currently in work**								
No	396	36.9	676	63.1	*0.482*	*1.00*		
Yes	137	39.0	214	61.0		*0.91 [0.71-1.17]*		
**BMI**								
*Mean - SD*	27.2	±4.4	28.4	±5.1	***<0.001***	***1.06 [1.03-1.08]***		
**Smoking habit**								
Never	332	40.0	499	60.0	***0.017***	*1.00*	**0.028**	*1.00*
Current smoker	83	41.3	118	58.7		*0.95 [0.69-1.29]*		*1.08[0.74-1.57]*
Ex-smoker	215	33.3	430	66.7		***1.33 [1.07-1.65]***		***1.40[1.09-1.80]***
**Family history of neoplasias**								
No	374	37.8	616	62.2	*0.952*	*1.00*		
Yes	220	37.9	360	62.1		*0.99[0.80-1.23]*		
**Family history of CRC**								
No	323	34.6	611	65.4	*0.212*	*1.00*		
Yes	59	39.9	89	60.1		*0.80[0.56-1.14]*		
**Specific signs and symptoms**								
Asymptomatic	40	25.0	120	75.0	***<0.001***	1.00		
Moderate signs and symptoms	132	44.0	168	56.0		***0.42[0.28-0.65]***		
Severe signs and symptoms	466	38.6	741	61.4		***0.53[0.36-0.77]***		
**Size of tumour**								
Locally small (T0-T1-T2)	96	25.5	280	74.5	***<0.001***	*1.00*	***<0.001***	*1.00*
Locally large (T3-T4)	555	41.4	787	58.6		***0.49[0.37-0.63]***		***0.51[0.37-0.69]***
**Lymph nodes**								
Absent	365	35.5	663	64.5	***0.015***	*1.00*		
Present	283	41.3	402	58.7		***0.78[0.64-0.95]***		
**Histological diagnosis**								
Adenocarcinoma	585	37.6	970	62.4	*0.597*	1.00		
Mucinous carcinoma	76	39.6	116	60.4		*0.92[0.68-1.25]*		
**Metastasis**								
Absent	523	35.3	960	64.7	***0.037***	1.00		
Present	63	44.1	80	55.9		**0.69[0.49-0.98]**		
**Differentiation**								
Low grade	490	36.8	843	63.2	*0.223*	1.00		
High grade	86	41.1	123	58.9		0.83[0.62-1.12]		
**Vascular invasion**								
Absent	475	37.7	784	62.3	*0.212*	1.00		
Present	98	42.1	135	57.9		0.83[0.63-1.11]		
**Perineural invasion**								
Absent	426	36.6	739	63.4	***0.001***	1.00		
Present	137	47.2	153	52.8		**0.64[0.50-0.83]**		
**Carcinoembryonic antigen (CEA)**								
Normal (0-5)	324	35.3	593	64.7	***0.010***	1.00		
Abnormal (>5)	188	42.5	254	57.5		**0.74[0.58-0.93]**		
**Cancer antigen 19-9**								
Normal (1-37)	219	35.3	401	64.7	***0.011***	1.00		
Abnormal (>37)	56	47.9	61	52.1		**0.59[0.40-0.89]**		
**Prior screening**								
No	547	41.1	783	58.9	***<0.001***	1.00	***<0.001***	1.00
Yes	89	25.4	262	74.6		**2.06[1.59-2.68]**		**1.79[1.32-2.43]**
**First line of treatment**								
Surgery	643	37.8	1056	62.2	*0.869*	1.00		
Chemotherapy	20	38.5	32	61.5		*0.97[0.55-1.72]*		
Radiotherapy	1	25.0	3	75.0		*1.83[0.19-17.6]*		

^*^ In multivariate logistic regression with a sample of 1,291 patients

**Table 4:** Bivariate and multivariate analysis with DFT in patients with rectal cancer

	≤30 days		>30 days		*Crude analysis*		Adjusted analysis*	
	n	%	p	OR95% CI	p	OR95% CI	p	OR95% CI
**Sex**								
Male	109	24.4	338	75.6	0.303	1.00		
Female	61	28.1	156	71.9		0.82[0.57-1.19]		
**Age**								
*Mean - SD*	65.9	±11.2	67.2	±11.0	0.168	1.01[0.99-1.03]		
**Marital status**								
Single	9	18.0	41	82.0	0.145	1.00		
Married-Cohabiting	111	27.3	295	72.7		0.58[0.27-1.24]		
Separated-Divorced	2	8.3	22	91.7		2.41[0.48-12.17]		
Widowed	18	26.9	49	73.1		0.60[0.24-1.47]		
**Education profile**								
No education-Primary education	97	23.3	320	76.7	**0.025**	1.00	**0.020**	1.00
Secondary-University	41	33.3	82	66.7		**0.61[0.39-0.94]**		**0.56[0.34-0.91]**
**Currently in work**								
No	107	25.4	314	74.6	0.845	1.00		
Yes	30	26.3	84	73.7		0.95[0.60-1.53]		
**BMI**								
*Mean - SD*	26.9	±4.1	27.1	±4.7	0.699	1.01[0.96-1.05]		
**Smoking habit**								
Never	71	25.5	207	74.5	0.989	1.00		
Current smoker	26	25.7	75	74.3		0.99[0.59-1.67]		
Ex-smoker	66	25.1	197	74.9		1.02[0.69-1.51]		
**Family history of neoplasias**								
No	88	25.2	261	74.8	0.757	1.00		
Yes	70	26.3	196	73.7		0.94[0.66-1.36]		
**Family history of CRC**								
No	91	25.2	270	74.8	0.386	1.00		
Yes	12	20.0	48	80.0		1.35[0.87-2.65]		
**Specific signs and symptoms**								
Asymptomatic	4	9.1	40	90.9	**0.009**	1.00	**0.031**	1.00
Moderate signs and symptoms	15	18.5	66	81.5		0.44[0.14-1.42]		0.67[0.16-2.74]
Severe signs and symptoms	146	28.2	371	71.8		**0.25(0.09-0.72)**		0.31[0.09-1.07]
**Size of tumour**								
Locally small (T0-T1-T2)	78	25.6	227	74.4	0.998	1.00		
Locally large (T3-T4)	88	25.6	256	74.4		1.00[0.70-1.42]		
**Lymph nodes**								
Absent	116	26.6	320	73.4	0.292	1.00		
Present	42	22.6	144	77.4		1.42[0.83-1.86]		
**Histological diagnosis**								
Adenocarcinoma	152	25.5	445	74.5	0.660	1.00		
Mucinous carcinoma	13	22.8	44	77.2		1.16[0.61-2.20]		
**Metastasis**								
Absent	138	24.0	436	76.0	0.751	1.00		
Present	10	26.3	28	73.7		0.89[0.42-1.87]		
**Differentiation**								
Low grade	106	23.2	351	76.8	0.175	1.00		
High grade	19	31.1	42	68.9		0.67[0.37-1.20]		
**Vascular invasion**								
Absent	127	25.1	378	74.9	0.490	1.00		
Present	9	20.5	35	79.5		1.31[0.61-2.79]		
**Perineural invasion**								
Absent	112	24.2	350	75.8	**0.042**	1.00	0.051	1.00
Present	29	34.9	54	65.1		**0.60[0.36-0.98]**		0.57[0.32-1.00]
**Carcinoembryonic antigen (CEA)**								
Normal (0-5)	105	25.5	306	74.5	0.722	1.00		
Abnormal (>5)	44	27.0	119	73.0		0.93[0.61-1.40]		
**Cancer antigen 19-9**								
Normal [1−37]	70	21.6	254	78.4	0.133	1.00		
Abnormal [>37]	14	31.8	30	68.2		0.59[0.30-1.17]		
**Prior screening**								
No	148	27.5	390	72.5	**0.025**	1.00		
Yes	15	16.3	77	83.7		**1.95[1.09-3.49]**		
**First line of treatment**								
Surgery	56	20.8	213	79.2	0.067	1.00		
Chemotherapy	76	29.0	186	71.0		0.64[0.43-0.96]		
Radiotherapy	38	28.6	95	71.4		0.66[0.41-1.06]		

^*^ In multivariate logistic regression with a sample of 433 patients

After adjusting for variables found to be statistically significant in the crude analysis, the multivariate analysis revealed the following to be independent protective factors against increased DFT: having university studies, for colon cancer [OR = 0.69; 95% CI 0.52-0.91] and for rectal cancer [OR = 0.56; 95% CI 0.34-0.91]; later tumour stage, for colon tumours, T3-T4, [OR = 0.51; 95% CI 0.37-0.69]; and for rectal tumours, the presence of severe [OR = 0.31; 95% CI 0.09-1.07] or moderate symptoms [OR = 0.67; 95% CI 0.16-2.74], compared with asymptomatic patients. However, DFT was greater in the patients with colon cancer who were ex-smokers [OR = 1.40; 95% CI 1.09-1.80] and in those who had had prior screening [OR = 1.79; 95% CI: 1.32-2.43] (Tables 3 and 4).

## DISCUSSION

Our study highlights the existence of delayed implementation of the first treatment among 65.5% of the population diagnosed with CRC. This finding lies within the 40-70% range of treatment delay previously reported [7].

Studies have been conducted to evaluate the prognostic influence of diagnostic and treatment delays on different types of cancer, and to determine the significant factors in this process. However, conflicting results have been obtained, due in part to differences in the characteristics of the populations analysed; furthermore, in most cases, the cohorts have been examined retrospectively and there have been differences in the time intervals studied [8]. This is a controversial issue, and it remains to be clarified. Unlike these earlier studies, our own research is based on a large number of patients recruited prospectively. We define excessive delay between diagnosis and treatment as a period exceeding 30 days, following previous recommendations and reports in this respect [9, 10, 11, 12, 13]

Unlike other studies on diagnostic and treatment delays in patients with CRC, our study population is distributed according to the location of the tumour (colon or rectal), in view of the well-known differences in the pathogenesis of each. We found DFT to be significantly greater for rectal tumours, as was also reported in the case of delay attributable to the patient [14]. Analysis of the delay according to the type of first treatment applied showed that this difference persisted when the first treatment was surgery, but not when it was chemotherapy or radiotherapy. This association is consistent with the findings of other studies, which have related the delay in surgical treatment for advanced stage (according to the Dukes system) rectal tumours, but not for tumours of the colon [15], probably because in localised and locally-advanced rectal tumours, and unlike for colon cancer, other diagnostic tests are required prior to treatment, such as pelvic magnetic resonance imaging and rectal endoscopic ultrasound examination [16]. Another difference between the two types of cancer was the relationship between DFT and the digestive symptoms diagnosed; a shorter DFT was only observed in patients with rectal cancer and moderate to severe symptoms, compared with mildly symptomatic or asymptomatic patients. Possibly the more pronounced and alarming symptoms resulting from rectal tumours, i.e. bleeding and pain, compared to the less specific and subacute ones provoked by colon tumours, lead patients with rectal cancer to seek a medical consultation at an earlier stage, thus expediting the diagnostic-therapeutic circuit. The physician prescribing the treatment will probably give preference to symptomatic patients, who are at increased risk of presenting complications from the tumour and therefore have a worse prognosis. It should also be taken into account that some patients with advanced tumours do not state the actual date of onset of their symptoms, or minimise it, due to a feeling of guilt at not having consulted the doctor sooner, and this too can exacerbate the DFT [17–19].

Studies of CRC have evaluated the relationship between tumour stage and diagnostic and therapeutic delays, and have found no association between these parameters [20]. Although some studies have shown that the DFT is shorter for patients presenting advanced stages of the disease [21], others have concluded the opposite [22]. Nevertheless, these conclusions cannot be generalised for tumours of the colon and rectum as if they were a single entity; on the contrary, they must be analysed independently, in view of the different natural history presented in each case [23, 24]. Thus, some retrospective studies have shown that advanced rectal tumours present an increased risk of DFT, in comparison with the initial stages, while no such differences were found for cancers of the colon [15]. On the other hand, in our own study, tumour stages T1-T2 experienced greater DFT than more advanced stages, but only in tumours of the colon. This difference might arise from the lower priority assigned to treatment for early-stage cancers, when symptoms are usually less apparent and hence delay the start of the therapeutic process. In a study of breast cancer, our group evaluated the different periods of delay, noting that higher tumour stages were associated with a shorter DFT, which was associated with a lower disease-free survival time. This outcome is probably produced by the priority granted by doctors to patients whose symptoms are more severe [6], which contradicts the traditional view that greater delay is associated with decreased survival time. This inverse correlation between treatment delay and survival has been described previously in studies of the endometrium and the lung [22, 25].

In our analysis of clinicopathological characteristics with known prognostic value and associated with increased tumour aggressiveness, the degree of histological differentiation and of lymphovascular invasion presented no relation to DFT. However, they were found to be related to distant metastases, lymph node involvement, perineural invasion and elevated tumour markers, all of which decrease the risk of severe DFT. However, when a multivariate analysis was performed, and other variables were taken into account, these differences did not persist, probably because the variables in question are more dependent on the biological behaviour of the tumour and on its intrinsic aggressiveness than on the period of treatment delay, as suggested by Symonds in a study of cervical cancer [26]. In other tumours, such as breast cancer, a significant association has also been described between the presence of more aggressive features and a shorter delay in initiating treatment; such features may include the non expression of hormone receptors, or non response to hormonal treatments in tumours that do express hormone receptors. These findings suggest that treatment may be expedited when the physician is aware of the extent of the tumour [6].

Among the sociocultural factors analysed, the lack of formal education or only having had primary education significantly increases the risk of DFT, for both rectal and colon tumours. Interestingly, this association, which has not received much previous research attention, influences DFT independently of other factors. One explanation for this might be that these patients do not understand the instructions received during the diagnosis-therapy process, and may also fail to keep the medical appointments necessary for a definitive tumour treatment to be undertaken. This population group, with a low cultural level, might also delay the start of treatment for fear of future treatments and distrust of the benefit derived from them. This possibility was raised in a recent study in which DFT was associated with a lack of knowledge of symptoms suggestive of cancer, and with the patient's unwillingness to visit the doctor, among other factors [27]. For these reasons, we believe that among certain population groups, with unhealthy living habits and a low educational profile, the risk of severe DFT is greater. In this respect, a retrospective study was conducted to obtain an ecological estimation of the socioeconomic status of patients with cancer (European Deprivation Index). No such relationship with DFT or with diagnostic delay was found, although it should be noted that this study included different types of cancer, with only 116 CRC [28].

Retrospective studies have evaluated social factors that might influence treatment delay, noting that black and/or elderly patients with rectal cancer were subject to greatest delay in initiating adjuvant chemotherapy [29]. In another study, of bowel cancer [30], elderly and/or unmarried patients were found to be most subject to this delay. Other studies evaluating prehospital delay have also found that lower socioeconomic level and lower education level are relevant factors. [14, 31].

Another feature of our population which the univariate analysis showed to be associated with increased treatment delay was a high BMI (>28) in patients with colon cancer. This relation would be explained, in part, by the complication of abdominal examination in the presence of a large pannus. One of the main causes of obesity in the West is an unhealthy living habit in terms of diet and exercise; this, too, is associated with a low socio-cultural level, which as mentioned previously is an independent predictor of treatment delay. The remaining demographic variables analysed–sex, age at diagnosis, family history of cancer, marital status and occupation–bore no significant relation with DFT.

The relationship between treatment delay and ex-smokers is a complex one. Elderly ex-smokers probably have more limitations of the respiratory function and require a larger number of tests before surgery. On the other hand, a patient who gives up smoking will probably believe him/herself at less risk of serious disease than a continuing smoker, and this factor, too, may influence communication with the doctor after diagnosis. In this respect, Mosher et al., in a study of patients diagnosed with lung cancer, reported that most ex-smokers rejected psychological therapy [32].

Our results show that a prior positive screening, in which faecal occult blood is detected, is associated with a greater risk of treatment delay; this relation has not been reported in previous studies. A priori, it seems illogical that a patient who has received CRC screening before any treatment is undertaken should suffer a delay for this reason. However, probably due to the person's asymptomatic state at the time of the consultation, no preference is expressed (unlike the case of a patient with manifest symptoms and at increased risk of complications from the tumour, requiring prompt treatment). Nevertheless, we considered the possible existence of confounding and of interaction with the other variables, and always obtained the same relationship between prior screening and subsequent treatment delay. Neither were there any interaction terms to be retained in the final model (data not shown).

Although it has been shown that delayed diagnosis and treatment does not appear to increase the risk of death in patients with symptomatic CRC, among the asymptomatic population early diagnosis and treatment may play a role in reducing morbidity and mortality [33]. The results presented should be considered with caution, and are subject to further analysis to determine whether, in the screened population, the greater delay observed impacts on survival.

The delay before cancer treatment is started is an important factor to be evaluated. This delay, which is a criterion of health care quality, should be prevented and reduced as far as possible in order to avoid the psychologically negative impact it may cause to patients. Numerous studies have shown that treatment delay is associated with certain clinical factors in CRC, but the present study is the first to establish that DFT depends not only on clinicopathological characteristics of the tumour, or on deficiencies of the healthcare system, but also on sociocultural characteristics of the population. We conclude, therefore, that more attention should be paid to health education regarding the initial symptoms related to this disease, especially among less educated social groups. The physician responsible for the patient's treatment, too, must be aware that these patients require special attention.

Finally, more multicentre studies should be conducted, in other countries and where different healthcare plans are used, in order to generalise the findings of our study. Another valuable area for future research would be to determine whether treatment delay also impacts on survival, as this association has not been clarified in recent reviews of the question [6, 34].

## MATERIALS AND METHODS

### Study design

This prospective, multicentre observational study was conducted in coordination with 22 public-sector hospitals in six regions of Spain (Andalusia, Canary Islands, Catalonia, Madrid, Valencia and the Basque Country) [35].

The patients were recruited prospectively and consecutively at each of the participating hospitals between June 2010 and December 2012. The study population included patients diagnosed with new colon or rectum cancer, stage I-IV and surgically treated, whether urgently or scheduled. All patients were included, whether or not they had previously received treatment, and a follow up study of five years was scheduled. Data were compiled directly from patients and also from their medical history.

### Study definitions

Excessive treatment delay was defined as an interval exceeding 30 days from pathological diagnosis to first treatment, in accordance with national guidelines and previous reports [10–13, 15]. First treatment was taken to be surgery, chemotherapy, radiotherapy, biological therapy or best supportive care. Date of diagnosis was the date when histological confirmation of the process was obtained, unless this coincided with the date of the intervention. In this case, we used as first date of diagnosis the suspected diagnosis [35].

The anatomical location of the tumour and the histology findings were coded in accordance with the International Classification for Oncology (ICD-O). Staging classification was based on the TNM recommendations of the International Union Against Cancer, 7th edition.

The following inclusion criteria were applied:

- Patients diagnosed with cancer of the colon (up to 15 cm above the anal margin) or of the rectum (between the anal margin and 15 cm above it), to which curative and/or palliative surgical treatment was applied for the first time.
- Signed informed consent provided.

The exclusion criteria were:

- Patients diagnosed with cancer of the colon or rectum in situ.
- Unresectable tumours.
- Mental or physical disorders that prevented the patient from answering the questionnaires.
- Terminal patients

The project was evaluated by the corresponding Research Committees and Clinical Research Ethics Committees at the hospitals. Informed consent was requested of the patients before surgery. Current legislative requirements regarding personal data (any information concerning individuals who were identified or identifiable) were followed at all times. All personal data were processed in such a way that the information obtained could not be associated with identified or identifiable persons (Protection of Personal Data Act, 15/1999, 13-12).

### Study variables

Data were compiled regarding the patients’ medical history: Sex, age, body mass index, prior screening, date of first contact with the hospital, first diagnosis, start of treatment, and the various types of first treatment considered (surgery, chemotherapy, radiotherapy, biological therapy or best supportive care). The date of diagnosis was taken as the date when the first histopathological report identifying the presence of cancer, was issued, except patients treated at the same time as they were diagnosed that we used as first date of diagnosis the suspected diagnosis date. The following laboratory and pathological factors were also recorded: tumour location (rectum or colon), degree of histological differentiation, tumour stage T and lymph node N (determined by the TNM clinical staging system), lymphovascular and perineural invasion, presence of metastasis, status of tumour markers such as carbohydrate antigen (*CA*) *19-9* and serial carcino-embryonic antigen (CEA). [36]

The following variables were self-reported by the patient: family history of colorectal cancer and other tumors, marital status, occupation at the time of the study, education profile, smoking habit and symptoms prior to surgery, date of onset of symptoms.

### Statistical design

A descriptive analysis was performed, with measures of central tendency and dispersion for the quantitative variables and frequency distributions for the qualitative ones. Differences were determined by bivariate analysis, segmenting by type of tumour and by time elapsed to first treatment, using the Student t test for quantitative variables and the chi-square test for qualitative ones. Finally, the treatment delay variable was used to perform a multivariate logistic regression analysis, using the variables with a value of p<0.1, together with the patient's age and sex. The level of statistical significance used in these analyses was p<0.05.

## References

[1] Verdecchia A, Francisci S, Brenner H, Gatta G, Micheli A, Mangone L, Kunkler I, Group EW Recent cancer survival in Europe: a 2000-02 period analysis of EUROCARE-4 data. Lancet Oncol.

[2] Andrews BT, Bates T Delay in the diagnosis of breast cancer: medico-legal implications. Breast.

[3] Esteva M, Ramos M, Cabeza E, Llobera J, Ruiz A, Pita S, Segura J, Cortes J, Gonzalez-Lujan L, DECCIRE research group Factors influencing delay in the diagnosis of colorectal cancer: a study protocol. BMC Cancer.

[4] Pita Fernández S, Pértega Díaz S, López Calviño B, González Santamaría P, Seoane Pillado T, Arnal Monreal F, Maciá F, Sánchez Calavera MA, Espí Macías A, Valladares Ayerbes M, Pazos A, Reboredo López M, González Saez L Diagnosis delay and follow-up strategies in colorectal cancer. Prognosis implications: a study protocol. BMC Cancer.

[5] Macià F, Pumarega J, Gallén M, Porta M Time from (clinical or certainty) diagnosis to treatment onset in cancer patients: the choice of diagnostic date strongly influences differences in therapeutic delay by tumor site and stage. J Clin Epidemiol.

[6] Redondo M, Rodrigo I, Pereda T, Funez R, Acebal M, Perea-Milla E, Jiménez E Prognostic implications of emergency admission and delays in patients with breast cancer. Support Care Cancer.

[7] Ramos M, Esteva M, Cabeza E, Campillo C, Llobera J, Aguiló A Relationship of diagnostic and therapeutic delay with survival in colorectal cancer: a review. Eur J Cancer.

[8] Ramos M, Esteva M, Cabeza E, Llobera J, Ruiz A Lack of association between diagnostic and therapeutic delay and stage of colorectal cancer. Eur J Cancer.

[9] Pérez G, Porta M, Borrell C, Casamitjana M, Bonfill X, Bolibar I, Fernández E, INTERCAT Study Group Interval from diagnosis to treatment onset for six major cancers in Catalonia, Spain. Cancer Detect Prev.

[10] Romero Gómez M, Alonso Redondo E, Borrego Dorado I, Briones Pérez de la Blanca E, Campos Rico A, Carlos Gil AM, de las Peñas Cabrera MD, del Río Urend S, Dotor Gracia M, Espinosa Bosch M, Fernández Ávila JJ, Fernández Echegaray R, Galindo Galindo A (2011). Cáncer colorrectal: proceso asistencial integrado. Sevilla- Consejería de Salud.

[11] Bleicher RJ, Ruth K, Sigurdson ER, Beck JR, Ross E, Wong YN, Patel SA, Boraas M, Chang EI, Topham NS, Egleston BL Time to Surgery and Breast Cancer Survival in the United States. JAMA Oncol.

[12] Guzman-Laura KP, Bolibar RI, Alepuz MT, Gonzalez D, Martin M Impact on the care and time to tumor stage of a program of rapid diagnosis and treatment of colorectal cancer. Rev Esp Enferm Dig.

[13] Bilimoria KY, Ko CY, Tomlinson JS, Stewart AK, Talamonti MS, Hynes DL, Winchester DP, Bentrem DJ Wait times for cancer surgery in the United States: trends and predictors of delays. Ann Surg.

[14] Mitchell E, Macdonald S, Campbell NC, Weller D, Macleod U Influences on pre-hospital delay in the diagnosis of colorectal cancer: a systematic review. Br J Cancer.

[15] Korsgaard M, Pedersen L, Sorensen HT, Laurberg S Treatment delay is associated with advanced stage of rectal cancer but not of colon cancer. Cancer Detect Prev.

[16] Glimelius B, Tiret E, Cervantes A, Arnold D, ESMO Guidelines Working Group Rectal cancer: ESMO Clinical Practice Guidelines for diagnosis, treatment and follow-up. Ann Oncol.

[17] Fernández E, Porta M, Malats N, Belloc J, Gallén M Symptom-to-diagnosis interval and survival in cancers of the digestive tract. Dig Dis Sci.

[18] Curless R, French J, Williams GV, James OF Comparison of gastrointestinal symptoms in colorectal carcinoma patients and community controls with respect to age. Gut.

[19] Roncoroni L, Pietra N, Violi V, Sarli L, Choua O, Peracchia A Delay in the diagnosis and outcome of colorectal cancer: a prospective study. European Journal of Surgical Oncology.

[20] Ramos M, Esteva M, Cabeza E, Llobera J, Ruiz A Lack of association between diagnostic and therapeutic delay and stage of colorectal cancer. Eur J Cancer.

[21] Kim IY, Kim BR, Kim YW Factors Affecting Use and Delay (>/=8 Weeks) of Adjuvant Chemotherapy after Colorectal Cancer Surgery and the Impact of Chemotherapy-Use and Delay on Oncologic Outcomes. PloS one.

[22] Myrdal G, Lambe M, Hillerdal G, Lamberg K, Agustsson T, Stahle E (2004). Effect of delays on prognosis in patients with non-small cell lung cancer. Thorax.

[23] Smith G, Carey FA, Beattie J, Wilkie MJ, Lightfoot TJ, Coxhead J, Garner RC, Steele RJ, Wolf CR Mutations in APC, Kirsten-ras, and p53--alternative genetic pathways to colorectal cancer. Proc Natl Acad Sci U S A.

[24] Richards MA, Westcombe AM, Love SB, Littlejohns P, Ramirez AJ Influence of delay on survival in patients with breast cancer: a systematic review. Lancet.

[25] Crawford SC, Davis J A, Siddiqui NA, De_Caestecker L, Gillis CR, Hole D, Penney G The waiting time paradox: population based retrospective study of treatment delay and survival of women with endometrial cancer in Scotland. BMJ.

[26] Symonds P, Bolger B, Hole D, Mao JH, Cooke T Advanced-stage cervix cancer: rapid tumour growth rather than late diagnosis. British journal of cancer.

[27] Abu-Helalah MA, Alshraideh HA, Da'na M, Al-Hanaqtah M, Abuseif A, Arqoob K, Ajaj A Delay in presentation, diagnosis and treatment for colorectal cancer patients in Jordan. J Gastrointest Cancer.

[28] Moriceau G, Bourmaud A, Tinquaut F, Oriol M, Jacquin JP, Fournel P, Magné N, Chauvin F Social inequalities and cancer: can the European deprivation index predict patients’ difficulties in health care access? a pilot study. Oncotarget.

[29] Cheung WY, Neville BA, Earle CC Etiology of delays in the initiation of adjuvant chemotherapy and their impact on outcomes for Stage II and III rectal cancer. Diseases of the colon and rectum.

[30] Hershman D, Hall MJ, Wang X, Jacobson JS, McBride R, Grann VR, Neugut AI Timing of adjuvant chemotherapy initiation after surgery for stage III colon cancer. Cancer.

[31] Macleod U, Mitchell ED, Burgess C, MacDonald S, Ramirez AJ Risk factors for delayed presentation and referral of symptomatic cancer: evidence for common cancers. Br J Cancer.

[32] Mosher CE, Winger JG, Hanna N, Jalal SI, Fakiris AJ, Einhorn LH, Birdas TJ, Kesler KA, Champion VL Barriers to mental health service use and preferences for addressing emotional concerns among lung cancer patients. Psychooncology.

[33] Pruitt SL, Harzke AJ, Davidson NO, Schootman M Do diagnostic and treatment delays for colorectal cancer increase risk of death?. Cancer Causes Control.

[34] Murchie P, Raja EA, Brewster DH, Campbell NC, Ritchie LD, Robertson R, Samuel L, Gray N, Lee AJ Time from first presentation in primary care to treatment of symptomatic colorectal cancer: effect on disease stage and survival. Br J Cancer.

[35] Quintana JM, Gonzalez N, Anton-Ladislao A, Redondo M, Bare M, Fernandez de_Larrea N, Briones E, Escobar A, Sarasqueta C, Garcia-Gutierrez S, Aguirre U, REDISSEC-CARESS/CCR group Colorectal cancer health services research study protocol: the CCR-CARESS observational prospective cohort project. BMC Cancer.

[36] Clinical practice guidelines for the use of tumor markers in breast and colorectal cancer. Adopted on May 17, 1996 by the American Society of Clinical Oncology. J Clin Oncol.

